# Nobiletin Alleviates Non-alcoholic Steatohepatitis in MCD-Induced Mice by Regulating Macrophage Polarization

**DOI:** 10.3389/fphys.2021.687744

**Published:** 2021-05-20

**Authors:** Si-wei Wang, Tian Lan, Hao Sheng, Fang Zheng, Mei-kang Lei, Li-xia Wang, Hang-fei Chen, Chun-yi Xu, Feng Zhang

**Affiliations:** ^1^Core Facility, The Quzhou Affiliated Hospital of Wenzhou Medical University, Quzhou People’s Hospital, Quzhou, China; ^2^Department of Pharmacy, The Quzhou Affiliated Hospital of Wenzhou Medical University, Quzhou People’s Hospital, Quzhou, China; ^3^Zhejiang University School of Medicine, Hangzhou, China; ^4^Department of Analytical Testing Center, Quzhou Customs, Quzhou, China; ^5^Agriculture and Rural Bureau of Changshan County, Quzhou, China; ^6^Zhejiang Chinese Medical University, Hangzhou, China

**Keywords:** nobiletin, NASH, RORα, macrophage polarization, KLF4

## Abstract

Non-alcoholic steatohepatitis (NASH) is an inflammatory disorder that is characterized by chronic activation of the hepatic inflammatory response and subsequent liver damage. The regulation of macrophage polarization in liver is closely related to the progression of NASH. The orphan nuclear receptor retinoic-acid-related orphan receptor α (RORα) and Krüppel-like factor 4 (KLF4) are key regulators which promote hepatic macrophages toward M2 phenotype and protect against NASH in mice. Nobiletin (NOB), a natural polymethoxylated flavone, is previously reported as a RORα regulator in diet-induced obese mice. However, it is still unclear whether NOB has the protective effect on NASH. In this study, we investigated the role of NOB in NASH using a methionine and choline deficient (MCD)-induced NASH mouse model. Our results showed that NOB ameliorated hepatic damage and fibrosis in MCD fed mice. NOB treatment reduced the infiltration of macrophages and neutrophils in the liver in MCD-fed mice. Of importance, NOB significantly increased the proportion of M2 macrophages and the expression of anti-inflammatory factors *in vivo* and *in vitro*. Meanwhile, NOB also decreased the population of M1 macrophages and the expression of proinflammatory cytokines. Mechanistically, NOB elevated KLF4 expression in macrophages. Inhibition of KLF4 abolished NOB regulated macrophage polarization. Furthermore, the regulation of NOB in KLF4 expression was dependent on RORα.

## Introduction

Non-alcoholic steatohepatitis (NASH), the progressive form of non-alcoholic fatty liver disease (NAFLD), is an inflammatory disorder that is characterized by liver inflammation, fibrosis and hepatocellular injury (Han et al., 2017; Kong et al., 2019; Xu et al., 2020). It has been known that macrophage plays a critical role in hepatic immune homeostasis by releasing cytokines and modulating immune cell response in NASH (Han et al., 2019). Macrophages, which can be activated in a number of ways, can be categorized as two main groups, designated M1 and M2 (Wang et al., 2019). The M1 macrophage phenotype is characterized by the production of high levels of pro-inflammatory cytokines such as tumor necrosis factor α (TNF-α) and interleukin 1β (IL-1β). In contrast, phenotypically M2 macrophages have been characterized as anti-inflammatory cytokines such as interleukin 10 (IL-10) and arginase-1 (Arg-1) (Murray, 2017). Increasing evidence has suggested that targeting M1/M2 polarization of macrophages could be effective strategy to alleviate NASH (Wan et al., 2014; Han et al., 2017, 2019; Kong et al., 2019).

Nuclear receptor retinoic-acid-related orphan receptor α (RORα), as a transcriptional factor, is involved in the regulation of various target genes related to lipid metabolism and inflammation (Jetten, 2009). It has been reported that RORα can directly regulate M1/M2 polarization switch of liver macrophages under the pathological conditions of NASH (Han et al., 2017). Importantly, RORα was low expression in liver of patients with NASH (Ou et al., 2013), and treatment with RORα activator could alleviate NASH (Han et al., 2014, 2017). These findings suggest that regulation of macrophage polarization by targeting RORα will provide an attractive strategy to limit NASH.

**FIGURE 1:**
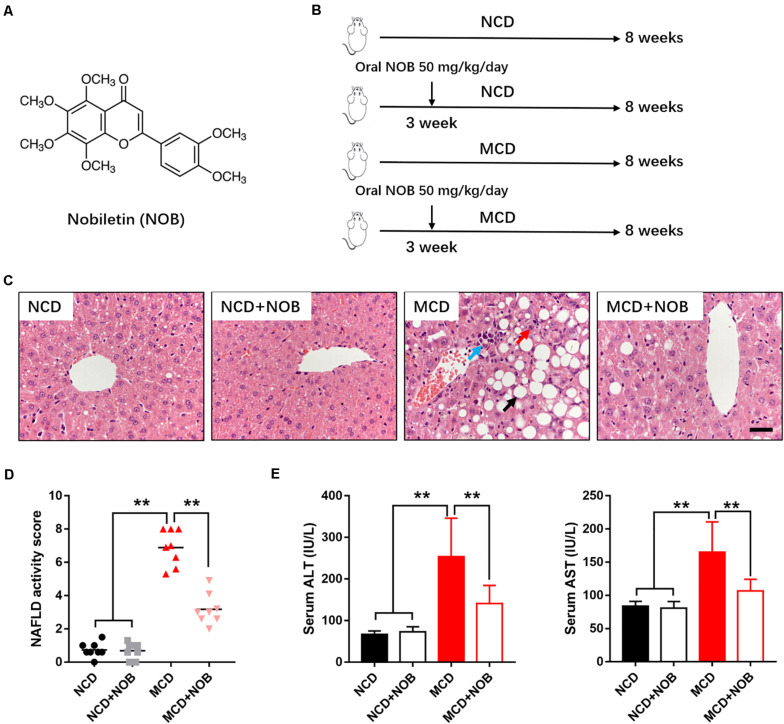
NOB ameliorates liver function in MCD fed mice. **(A)** The chemical structure of NOB. Its molecular weight is 402.39. **(B)** The schematic diagram for MCD-induced NASH and NOB administration. C57BL/6 mice were fed either a chow diet as normal control (NCD) or the MCD diet for 8 weeks to induce NASH (MCD). Mice were treated with daily oral doses of NOB (50 mg/kg) from the third week of MCD diet feeding. Water was gavaged as control. **(C)** The representative images of H&E staining in livers from each group. Scale bar = 300 μm. Black arrow denotes macrovesicular steatosis; red arrow denotes hepatocellular ballooning; blue arrow denotes lobular inflammation. **(D)** The NAFLD activity score was shown. **(E)** Serum alanine aminotransferase (ALT) and aspartate aminotransferase (AST) levels from each group. Data were expressed as the mean ± SD (*n* = 8). **P < 0.01.

Nobiletin (5,6,7,8,3′,4′-hexamethoxyflavone, NOB) (Figure 1A) is a natural polymethoxylated flavone extracted exclusively in citrus fruits (Di Donna et al., 2013). It has a wide range of beneficial properties, including anti-obesity (Morrow et al., 2020), anti-diabetic (Mulvihill et al., 2011), anti-inflammatory (Rong et al., 2021), anti-aging (Yang et al., 2020), and anti-tumor (Feng et al., 2020) activities. Previous studies have shown that NOB is a RORα regulator. It can activate RORα to protect against metabolic dysfunctions (He et al., 2016; Nohara et al., 2019). However, the direct effect of NOB on NASH has not been reported, and it is also unclear that whether NOB could regulate macrophage polarization to alleviate NASH by activating RORα.

In current study, we thus evaluated the effect of NOB on NASH using a methionine and choline deficient (MCD)-induced mouse model, and we investigated its effects and molecular mechanism on macrophage polarization *in vivo* and *in vitro*.

## Materials and Methods

### Chemicals and Reagents

Nobiletin (NOB, CAS# 478-01-3, HPLC ≥ 98%), and SR3335 (CAS# 293753-05-6) were purchased from Shanghai Yuanye Biological Technology, China. Recombinant Murine Interleukin-4 (IL-4, #C600050) was purchased from Sangon Biotech, Shanghai, China. Lipopolysaccharide (#L2630) was purchased from Sigma, United States.

### Animal Experiments

All animal experiments were performed in accordance with the Experimental Animal Center of Zhejiang University of Traditional Chinese Medicine, China. Experimental procedures were approved by the Ethics Committee of Animal Experiments of Quzhou people’s hospital, China. Male C57BL/6 mice (6–8 weeks, specified pathogen free) were purchased from GemPharmatech Co., Ltd., Jiangsu, China (license number of animal production: SYXK2015-0001). All animals were housed in room temperature, allowed free access to distilled water and common pelleted food, and placed under a 12-h/12-h dark/light cycle.

After 1 week of adaptation, the mice were randomly divided into four groups (*n* = 8/group).

• NCD group: These mice were fed with a control diet for methionine and choline deficient L-Amino acid diet (Research diet A02082003B, Research Diet, NJ) and received intragastrically administered distilled water.
• NCD + NOB group: These mice were fed with a control diet for methionine and choline deficient L-Amino acid diet (Research diet A02082003B, Research Diet, NJ) and treated with 50 mg/kg per day of intragastrically administered NOB.
• MCD group: These mice were received a methionine and choline deficient L-Amino acid diet (Research diet A02082002BR, Research Diet, NJ) and received intragastrically administered distilled water.
• MCD + NOB group: These mice were fed with a methionine and choline deficient L-Amino acid diet (Research diet A02082002BR, Research Diet, NJ) and treated with 50 mg/kg per day of intragastrically administered NOB.

As described, the NOB administration started from the third week and the experiment lasted for 8 weeks in total (Figure 1B).

### Biochemical Testing

Serum alanine aminotransferase (ALT), and aspartate aminotransferase (AST) were detected by biochemical analyzer according to the manufacturer’s instruction (DiaSys Diagnostic Systems, Shanghai, China).

### Histopathological Analysis

Liver tissues were fixed with 10% formalin, embedded in paraffin, sectioned, and stained with hematoxylin and eosin (H&E) and Sirius red. NAFLD activity score (NAS) was graded in a blinded manner according to our previously described (Wang et al., 2020a). Mean scores were evaluated through calculating 5 different 400 × microscopic fields per mouse section by two independent trained observers.

Fibrosis score based on the Kleiner/Brunt criteria adapted to rodents (0, no fibrosis; 1, focal pericellular fibrosis in zone 3; 2, perivenular and pericellular fibrosis confined to zones 2 and 3; 3, bridging fibrosis; 4, cirrhosis) (Amrutkar et al., 2016). The area of Sirius red stained was calculated in 5 randomly selected 200 × microscopic fields per mouse section using ImageJ software (U.S. National Institutes of Health, Bethesda, MD).

### Immunohistochemical Staining

Deparaffinized and blocked 5 μm sections were incubated with anti-α-SMA (1: 200, # 19245, Cell Signaling Technology), anti-F4/80 (1: 200, # 2370, Cell Signaling Technology) and anti-CD68 (1: 100, ab125212, Abcam) using MaxVision HRP-Polymer anti-Rabbit IHC Kit (MXB Biotechnologies, Fuzhou, China) to develop signal. The area of positive stained was calculated in 5 randomly selected 200× or 400× microscopic fields per mouse section using ImageJ software (U.S. National Institutes of Health, Bethesda, MD).

### Immunofluorescence Staining and Analysis

Deparaffinized and blocked 5 μm sections were permeabilized in 0.2% Triton X-100 for 15 min and blocked at room temperature with 3% BSA for 1 h. Primary antibodies used were CD32 (1: 200, cat. no. sc-166711, Santa Cruz Biotechnology) and CD206 (1: 200, cat. no. ab8918, Abcam). After three washes with PBS, the sections incubated with Alexa Fluor 488 goat anti-rabbit IgG for 1 h at room temperature. The fluorescence was visualized by a SUNNY RX50 fluorescence microscope. The number of immunofluorescent positive cells was counted using ImageJ software (U.S. National Institutes of Health, Bethesda, MD).

### Cell Culture

RAW 264.7 and 293T cell line were all obtained from the Shanghai Bank of Cell Lines (Shanghai, China). RAW 264.7 cells were cultured in RPMI-1640, and 293T cells were in DMEM. All these were supplemented with 10% fetal bovine serum (FBS, Gibco, United States), 100 U/mL penicillin, and 100 U/mL streptomycin at 37°C in a humidified atmosphere with 5% CO_2_. The source of the cell line was identified by STR profiling and tested for mycoplasma contamination.

### RNA Isolation and Quantitative RT-PCR

Total RNA was extracted from liver tissues and cells using TRIzol reagent (#DP424, Tiangen Biotech Co., Ltd., Beijing, China). cDNA was synthesized by reverse transcriptase kits (ThermoFisher Scientific, Waltham, MA) according to the manufacturer’s instructions. Quantitative real-time PCR was performed using SGExcel FastSYBR Mixture (#B532955-0005, Sangon Biotech Co., Ltd., Shanghai, China) on Roche LightCycler^*R*^ 480 Quantitative PCR System (Indianapolis, United States). The relative gene expression levels were calculated using the 2^–ΔΔ*Ct*^ method. Primers used are listed in Table 1.

**TABLE 1:** The primers used in this study for real time PCR.

**Description**	**Sense primer (5′→3′)**	**Antisense primer (5′→3′)**
*Il-6*	CTGCAAGAGACTTCCATCCAG	AGTGGTATAGACAGGTCTGTTGG
*Il-1*β	TTCAGGCAGGCAGTATCACTC	GAAGGTCCACGGGAAAGACAC
*Tnf-*α	CTGAACTTCGGGGTGATCGG	GGCTTGTCACTCGAATTTTGAGA
*Klf4*	GGCGAGTCTGACATGGCTG	GCTGGACGCAGTGTCTTCTC
*Cyp7b1*	GGAGCCACGACCCTAGATG	GCCATGCCAAGATAAGGAAGC
*Glut2*	TCAGAAGACAAGATCACCGGA	GCTGGTGTGACTGTAAGTGGG
*Gck*	TGAGCCGGATGCAGAAGGA	GCAACATCTTTACACTGGCCT
*Gapdh*	TGAGGCCGGTGCTGAGTATGT	CAGTCTTCTGGGTGGCAGTGAT

### ChIP Assay

For ChIP assay, we used enzymatic ChIP kit from Cell Signaling (Cat. #9003) according to the manufacturer’s instruction. Briefly, cells were crosslinked with 1% formaldehyde for 10 min at room temperature. Then, the reaction was stopped using 125 mM glycine. The chromatin was enzymatically digested by micrococcal nuclease at 37°C for 20 min. The digested chromatin was then briefly sonicated to break nuclear membranes. After preclearing, chromatin (500 μg) was subjected to immunoprecipitation with anti-RORα (1: 50, PA1-812, ThermoFisher Scientific) antibody or with anti-IgG as negative control at 4°C overnight. The protein-DNA complexes were then separated by incubation with protein G beads. The magnetic beads were then washed using buffers supplied with the kit to acquire chromatin. The eluted DNA was purified and analyzed by real-time PCR with specific primers: Sense, 5′- CAGAGTTAAACTGGCCTAGTTCCA-3′; Antisense, 5′-CTTTCTCTTGGTTTTGGCAGAGGA-3′. The primer sequences for ChIP-qPCR were obtained from previous publication (Han et al., 2017).

### siRNA and Transfection

siRNAs used for KLF4 knockdown were ordered from GenePharma (Shanghai, China). Transfection of siRNAs to Raw264.7 was performed using Lipofectamine RNAiMAX (#13778-075, ThermoFisher Scientific) according to manufacturer’s instruction.

### Statistical Analysis

GraphPad Prism 7 software (GraphPad Software, La Jolla, CA) was used for all statistical analyses. All results are presented as means ± SD. Student’s unpaired two-tailed *t*-test was using for comparisons between two groups. The one-way or two-way analysis of variance (ANOVA) followed by Tukey’s multiple comparison’s test was applied for comparisons between more than two groups. Differences were considered to be statistically significant at *P* < 0.05and highly significant at *P* < 0.01.

## Results

### NOB Ameliorates Liver Function in MCD Fed Mice

To estimate the effect of NOB on NASH, we established MCD-induced NASH mouse model (Figure 1B). Microscopic examination of H&E-stained liver sections revealed that MCD diet triggered marked macrovesicular steatosis, hepatocellular ballooning and lobular inflammation in mice; however, administration of NOB ameliorates these pathological changes (Figure 1C). NOB also reduced NAFLD activity score (NAS) (Figure 1D). In addition, NOB treatment decreased serum ALT and AST levels in MCD fed mice (Figure 1E), suggesting improved liver function.

### NOB Attenuates Liver Fibrosis in MCD-Induced Mice

**FIGURE 2:**
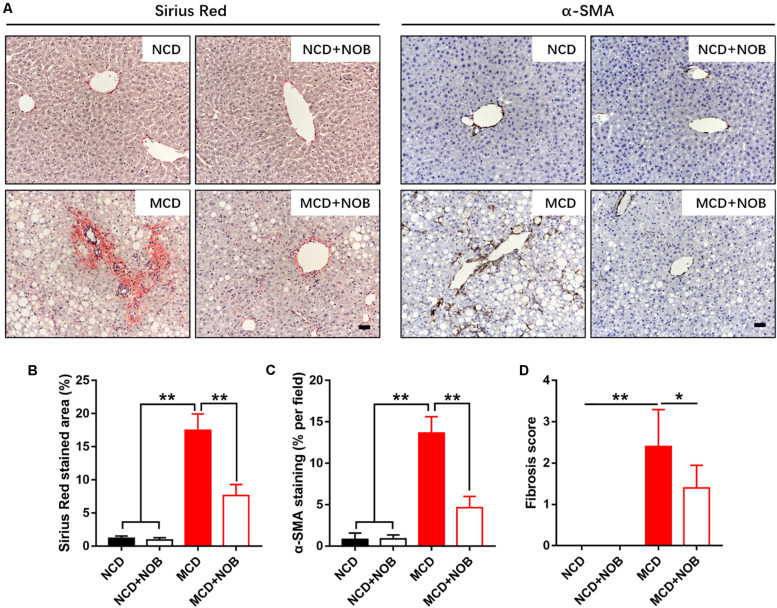
The effect of NOB on liver fibrosis in MCD-induced mice. The deposited collagen and activated HSCs were shown by Sirius red and α-SMA staining, respectively. **(A)** The representative images of Sirius red staining and α-SMA immunohistochemical staining in liver from each group. Scale bar = 300 μm. **(B)** Quantification and statistical analysis of sirius red-stained areas was shown. **(C)** Quantification and statistical analysis of α-SMA-stained areas was shown. **(D)** Quantification of fibrosis score was shown. Data were expressed as the mean ± SD (*n* = 8). *P < 0.05, **P < 0.01.

NASH is a progressive form of NAFLD where inflammation causes liver damage and fibrosis (Wattacheril et al., 2018). Next, we explored the impact of NOB treatment in hepatic fibrosis of MCD-fed mice. Our result showed that NOB reduced MCD-induced perivenular fibrosis, as revealed by picrosirius red staining for collagen fibers (Figures 2A,B). Immunohistochemical staining for α-SMA, a marker for activated hepatic stellate cells (HSCs), showed that NOB treatment obviously decreased activated HSCs in MCD fed mice (Figures 2A,C). Furthermore, the administration of NOB obviously lowered the fibrosis score in MCD fed mice compared to their counterparts (Figure 2D).

### NOB Suppresses the Infiltration of Macrophages and Neutrophils in MCD Fed Mice

**FIGURE 3:**
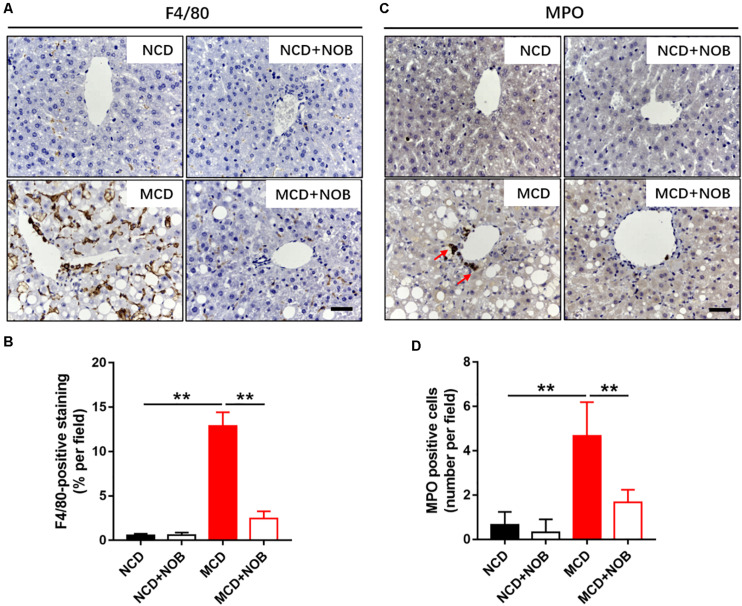
The effect of NOB on the infiltration of macrophages and neutrophils in MCD fed mice. **(A)** The representative images of F4/80 immunohistochemical staining in liver from each group. Scale bar = 300 μm. **(B)** Quantification and statistical analysis of F4/80-stained areas was shown. Data were expressed as the mean ± SD (*n* = 8). **(C)** The representative images of myeloperoxidase (MPO) immunohistochemical staining in liver from each group. Scale bar = 300 μm. Red arrow points to MPO-positive cells. **(D)** Quantification and statistical analysis of MPO-positive cells in liver from each group. Data were expressed as the mean ± SD (*n* = 8). **P < 0.01.

Inflammation is a key feature of NASH and has been found to be crucial for fibrogenesis (Browning and Horton, 2004; Bataller and Brenner, 2005). Macrophages and neutrophils are important inflammatory cells in NASH (Wattacheril et al., 2018). F4/80 is frequently used marker for identification of macrophage population (Wang et al., 2019, 2020b), and neutrophils are characterized by high expression of myeloperoxidase (MPO) (Klebanoff, 2005; Wang et al., 2020a). Feeding with MCD diet induced overt gathering of F4/80 positive macrophages, which was reduced by NOB treatment (Figures 3A,B). Administration of NOB also significantly reduced the elevated hepatic MPO^+^ cells in mice fed with MCD diet (Figures 3C,D). Notably, it was obvious that the population of neutrophils was smaller than the macrophages in MCD-induced mice (Figures 3A,C), suggesting a dominant role of macrophages in this model of NASH.

### NOB Decreases M1 Macrophages and the Expression of Proinflammatory Cytokines *in vivo* and *in vitro*

**FIGURE 4:**
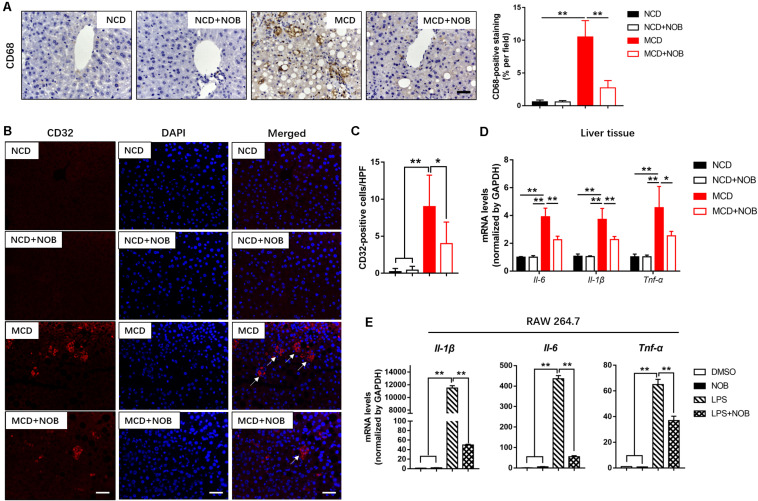
The effect of NOB on M1 macrophage polarization and expression of inflammatory properties in liver tissue and RAW 264.7 cells. **(A)** Left, the representative images of CD68 immunohistochemical staining in liver from each group. Scale bar = 300 μm. Right, quantification and statistical analysis of CD68-stained areas. Data were expressed as the mean ± SD (*n* = 8). **(B)** Representative fluorescence microscopic images of CD16/32-stained liver sections. Scale bar = 300 μm. **(C)** Quantification and statistical analysis of CD16/32-positive cells in liver tissue. We measured the number of CD16/32-positive membranes from at least five high power fields (HPF, original magnification, 400×). **(D)** The mRNA levels of *Il-6*, *Il-1*β and *Tnf-*α in livers of MCD-induced mice were evaluated using RT-qPCR. Data were expressed as the mean ± SD (*n* = 5). **(E)** RAW 264.7 cells were pretreated with DMSO, NOB (100 μM), LPS (200 ng/mL), or LPS (200 ng/mL) + NOB (100 μM) for 16 h. The expression of proinflammatory factors *Il-6*, *Il-1*β, and *Tnf-*α determined by RT-qPCR. Values are expressed as mean ± SD (*n* = 3). *P < 0.05, **P < 0.01.

Macrophages are heterogeneous population and include two major subsets: “proinflammatory” M1 and “immunoregulatory” M2 macrophages, according to their phenotype and functions in inflammatory responses (Wan et al., 2014; Kong et al., 2019). Polarization of macrophages toward the M1 phenotype would accelerate disease progression of NASH (Kong et al., 2019). As shown in Figures 4A–C, MCD diet increased hepatic M1 macrophages in mice, as detected by immunofluorescence staining with antibodies against the M1 macrophage marker CD32 and CD68 (Wang et al., 2019). NOB treatment markedly decreased the number of CD32 or CD68-postive M1 macrophages in MCD fed mice (Figures 4A–C). Consistently, NOB significantly inhibited the expression of proinflammatory cytokines associated with M1 macrophage such as *Il-6*, *Il-1*β, and *Tnf-*α in livers of MCD fed mice and LPS treated RAW 264.7 cells (Figures 4D,E). These results indicate that NOB inhibits macrophage polarization toward M1 subtype.

### NOB Increases the Population of M2 Macrophages and the Expression of Anti-inflammatory Factors *in vivo* and *in vitro*

**FIGURE 5:**
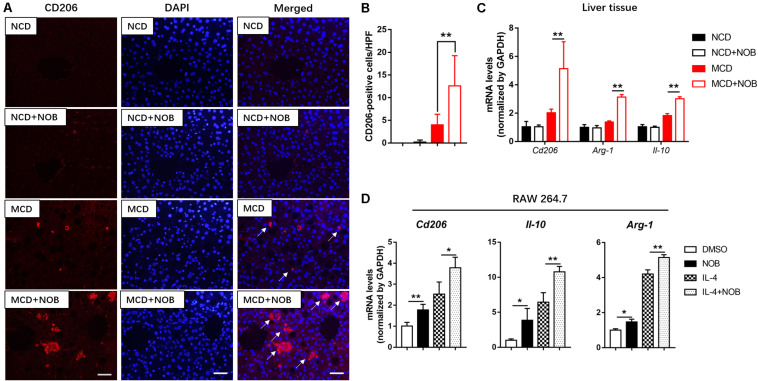
The effect of NOB on M2 macrophage polarization and the expression of anti-inflammatory factors in liver tissue and RAW 264.7 cells. **(A)** Representative fluorescence microscopic images of CD206-stained liver sections. Scale bar = 300 μm. **(B)** Quantification and statistical analysis of CD206-positive cells in liver tissue. We measured the number of CD206-positive membranes from at least five high power fields (HPF, original magnification, 400×). **(C)** The mRNA levels of *Cd206*, *Arg-1*, and *Il-10* in the livers of MCD-induced mice were evaluated using RT-qPCR. Data were expressed as the mean ± SD (*n* = 5). **(D)** RAW 264.7 cells were pretreated with DMSO, NOB (100 μM), LPS (200 ng/mL), or LPS (200 ng/mL) + NOB (100 μM) for 16 h. The mRNA levels of *Cd206*, *Arg-1*, and *Il-10* were determined by RT-qPCR. Values are expressed as mean ± SD (*n* = 3). *P < 0.05, **P < 0.01.

M2 macrophages promote resolution of inflammation and protect hepatocytes against NASH (Wan et al., 2014). CD206 is considered as a marker of M2 macrophages (Han et al., 2017; Wang et al., 2019). As shown in Figures 5A,B, the number of M2 macrophages was significantly increased after NOB treatment in the livers of MCD fed mice by CD206 immunofluorescence staining, compared to their MCD-diet-only counterparts. Additionally, the results of RT-qPCR also confirmed that NOB increased the expression of M2 markers *Cd206*, *Il-10* and *Arg-1* in liver of MCD-induced mice (Figure 5C). IL-4 is a strong inducer of M2 polarity in macrophages (Patel et al., 2017). Our results showed that NOB enhanced M2 polarization and the expression of M2 markers *Cd206*, *Il-10*, and *Arg-1* in IL-4 free or treated RAW 264.7 cells (Figure 5D).

### KLF4 Is Required for the Regulation of NOB on Macrophage Polarization

**FIGURE 6:**
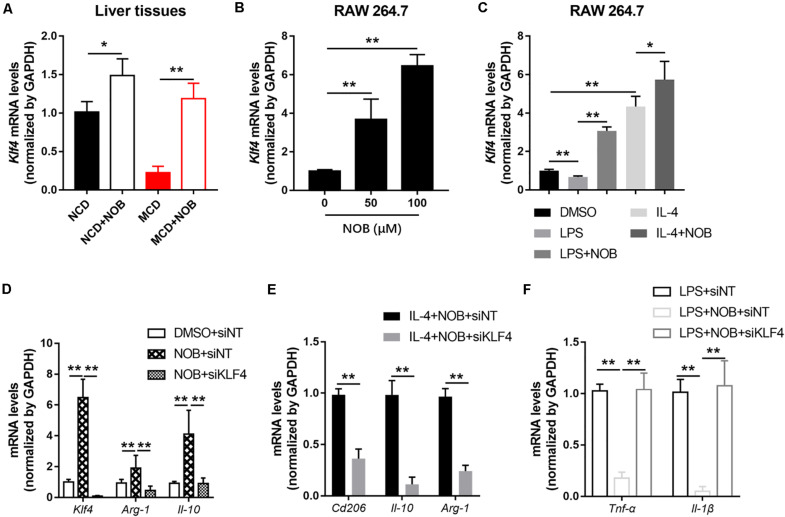
NOB promotes M2 polarization through increasing *Klf4* expression. **(A)** The mRNA expression of *Klf4* in the livers of MCD-fed mice was evaluated by RT-qPCR. Data were expressed as the mean ± SD (*n* = 5). **(B)** RAW 264.7 cells were treated with DMSO, NOB (50 μM) or NOB (100 μM) for 16 h. The mRNA expression of *Klf4* was determined by RT-qPCR. **(C)** RAW 264.7 cells were pretreated with DMSO, LPS (200 ng/mL), LPS (200 ng/mL) + NOB (100 μM), IL-4 (20 ng/mL), or IL-4 (20 ng/mL) + NOB (100 μM) for 16 h. The mRNA expression of *Klf4* was measured by RT-qPCR. **(D)** RAW 264.7 cells were transiently transfected with control (siNT) or KLF4 (siKLF4) siRNA and treated with either DMSO or NOB for 16 h as indicated. The mRNA levels of *Klf4*, *Arg-1*, and *Il-10* were determined by RT-qPCR. **(E)** The siRNA-NT or siRNA-KLF4 cells were treated with IL-4 (20 ng/mL) + NOB (100 μM) for 16 h. The mRNA levels of *Cd206*, *Arg-1*, and *Il-10* were measured by RT-qPCR. **(F)** The siRNA-NT cells were treated with LPS (2,00 ng/mL) or LPS (200 ng/mL) + NOB (100 μM). The siRNA-KLF4 cells were treated with LPS (200 ng/mL) + NOB (100 μM) for 16 h. The mRNA levels of *Il-1*β and *Tnf-*α were quantified by RT-qPCR. Values are expressed as mean ± SD (*n* = 3). *P < 0.05, **P < 0.01.

Krüppel-like factor 4 (KLF4) is a critical regulator of macrophage polarization (Liao et al., 2011). It cooperated with STAT6 to induce an M2 genetic program and inhibit M1 polarization. Macrophage KLF4 expression was robustly induced in M2 macrophages and strongly reduced in M1 macrophages (Liao et al., 2011; Murray, 2017). We found that NOB treatment increased *Klf4* expression both in NCD and MCD fed mice, especially in MCD group (Figure 6A). Furthermore, NOB treatment elevated *Klf4* expression *in vitro* in a dose dependent manner (Figure 6B). NOB also reversed the LPS induced inhibition on *Klf4* expression and further enhanced IL-4-induced *Klf4* expression in RAW 264.7 (Figure 6C). To determine whether KLF4 is indispensable to NOB mediated polarization of macrophages, we depleted KLF4 via siRNA in RAW 264.7 cells (Figure 6D). As shown in Figure 6D, NOB induced expression of *Il-10* and *Arg-1* was completely abolished by siKLF4. Knockdown of KLF4 also reduced the expression of NOB upregulated M2 marker genes (*Cd206*, *Il-10*, and *Arg-1*) in IL-4-treated RAW 264.7 cells (Figure 6E). Conversely, the inhibitory effect of NOB on the expression of M1 markers (*Il-1*β and *Tnf-*α) in LPS induced RAW 264.7 cells was counteracted by KLF4 knockdown (Figure 6F). These results indicate that NOB-mediated macrophage polarization is dependent on KLF4.

### The NOB-Induced Klf4 Expression and Macrophage Polarization Are RORα Dependent

**FIGURE 7:**
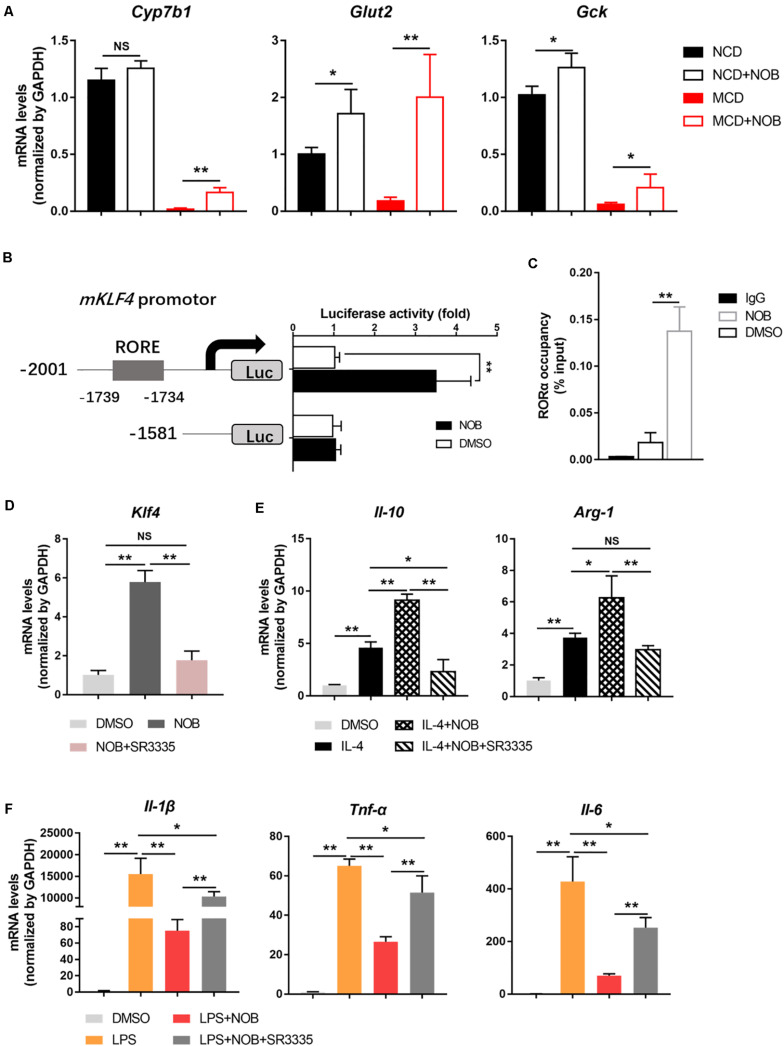
NOB induces *Klf4* expression and macrophage polarization through activiting RORα. **(A)** RT-qPCR analysis of RORα target genes *Cyp7b1*, *Glut2*, and *Gck* in the livers of MCD-fed mice. Data were expressed as the mean ± SD (*n* = 5). **(B)** Schematic diagram of the mouse Klf4 promoter with the RORα binding element (RORE) shown as gray box. 293T cells were transfected with luciferase reporters driven by Klf4 promoter with or without RORE and treated with DMSO or NOB (100 μM) for 8 h. **(C)** RAW 264.7 cells were treated with DMSO or NOB (100 μM) for 16 h. The binding of RORα to *Klf4* promotor was assessed by ChIP-qPCR. **(D)** RAW 264.7 cells were treated with DMSO, NOB (100 μM) or SR3335 (10 μM) for 16 h. The mRNA level of *Klf4* determined by RT-qPCR. **(E)** RAW 264.7 cells were treated with DMSO, IL-4 (20 ng/mL), IL-4 (20 ng/mL) + NOB (100 μM) or IL-4 (20 ng/mL) + NOB (100 μM) + SR3335 (10 μM) for 16 h. The mRNA levels of *Arg-1* and *Il-10* determined by RT-qPCR. **(F)** RAW 264.7 cells were pretreated with DMSO, LPS (200 ng/mL), LPS (200 ng/mL) + NOB (100 μM) or LPS (200 ng/mL) + NOB (100 μM) + SR3335 (10 μM) for 16 h. Proinflammatory factors *Il-6*, *Il-1*β, and *Tnf-*α determined by RT-qPCR. Values are expressed as mean ± SD (*n* = 3). *P < 0.05, **P < 0.01.

It is reported that the expression of KLF4 is regulated by orphan nuclear receptor retinoic-acid-related orphan receptor α (RORα) in hepatic macrophages (Han et al., 2017). In addition, NOB is known as an RORα activator (He et al., 2016; Nohara et al., 2019). Therefore, we investigated whether RORα is involved in NOB-mediated Klf4 expression and macrophage polarization. First, we found that NOB induced the expression of RORα target genes (*Cyp7b1*, *Glut2*, and *Gck*) in the livers of both NCD and MCD fed mice, implying RORα was activated by NOB *in vivo* (Figure 7A). To further explore the underlying mechanism, we expressed luciferase reporters driven by mouse Klf4 promotor in 293T cells. As shown in Figure 7B, NOB efficiently induced luciferase activity in cells transfected with wild type but not RORE deleted Klf4 promotor, suggesting the binding of RORα to ROR element in *Klf4* promotor was required to NOB mediated *Klf4* expression. Furthermore, we determined whether NOB promoted the transcriptional activity of endogenous RORα by ChIP-qPCR using RORα specific antibody. As expected, NOB treatment largely enhanced the binding of RORα to *Klf4* promotor in RAW 264.7 cells (Figure 7C). Moreover, NOB-induced Klf4 expression in RAW 264.7 cell was significantly inhibited by SR3335, an inverse agonist of RORα (Han et al., 2017; Figure 7D). SR3335 also abolished NOB-mediated upregulation of M2 markers (*Il-10* and *Arg-1*) in IL-4 treated RAW 264.7 (Figure 7E). Furthermore, the inhibition of M1 markers (*Il-6*, *Il-1*β, and *Tnf-*α) after NOB treatment in LPS challenged RAW 264.7 was partially reversed by SR3335 (Figure 7F). In general, these results suggest that NOB-mediated macrophage polarization is RORα dependent.

## Discussion

In current study, we evaluated the effect of NOB on NASH using MCD-induced mouse model. We observed that NOB administration ameliorated hepatic inflammation, reduced activated hepatic stellate cells and liver fibrosis. Besides, we found M2 type macrophages were significantly increased in the livers of NOB treated NASH mice accompanied by elevated expression of anti-inflammatory cytokine IL-10. Mechanistically, the activation of RORα by NOB was required for the enhanced M2 polarization. Inhibition of RORα activity by small molecular inhibitor abolished the effects of NOB on the induction of *Klf4* and *Il-10* expression in RAW 264.7 cells.

Previous study has shown that activating RORα can promote the M2 polarization of macrophages by up-regulating the expression of *Klf4* (Odegaard et al., 2008). In this study, we found that NOB promoted the M2 alternative activation of RAW 264.7 by activating RORα *in vitro*. Although we could not determine whether it is the case *in vivo* by tracing the fate of individual macrophage, our data still provided some hints. The population of F4/80-positive macrophages in the livers of MCD fed mice was obviously reduced after NOB treatment, even in this case, the macrophages with M2 markers were remarkably increased compared to the control group, suggesting enhanced M2 polarization also occurred *in vivo*.

In the experiments in mouse model of NASH, M1 macrophages with a pro-inflammatory phenotype seem to worsen the disease. Different mouse strains are innately prone to different immune responses after feeding with NASH diet (Mills et al., 2000). M1-prone C57BL/6 mice fed the MCD diet showed an increased tendency toward steatosis and hepatic inflammation compared to the M2 prone BALB/c mice (Maina et al., 2012; Kazankov et al., 2019). In contrast to M1 macrophages, M2 macrophages with an anti-inflammatory phenotype have been associated with ameliorated hepatic injury in NAFLD and improved insulin sensitivity (Odegaard et al., 2008; Wan et al., 2014). Pharmacological alteration of macrophage polarization toward an M2 phenotype could partially reverse hepatic steatosis and hepatocyte apoptosis induced by HFD feeding (Kazankov et al., 2019). Of interest, the conditioned medium from IL-4 induced M2-type macrophages promotes apoptosis of M1-type macrophages *in vitro*. Mechanistically, IL-10 released from M2 macrophages induces high inducible nitric oxide synthase-expressing M1 Kupffer cell (KC) death through paracrine activation of the enzyme arginase (Wan et al., 2014).

In this study, we observed that in mice fed with MCD diet, administration of NOB also reduced MPO positive monocytes. Although the relative number of monocytes is significantly different between NOB treated and the control group, the absolute number of monocytes even in the control group feeding with MCD diet is far less than that of macrophages. It is supposed that most monocytes are transformed into macrophages in this case. It is well established that in addition to liver-resident Kupffer cells, monocyte derived macrophages have a major role in the pathogenesis of NAFLD and NASH. Whether NOB have different effects on these two types of macrophages derived from different sources is an open question worthy of attention in the follow-up research.

In our previous study, we reported another bioactive flavone hesperetin could inhibits hepatic inflammation via AMPK/CREB/SIRT1 pathway (Wang et al., 2020b). We found hesperetin increased SIRT1 expression through activating AMPK. Besides, AMPK can also elevate SIRT1 activity by enhancing the mitochondrial metabolism and thereafter increasing the abundance of intracellular NAD^+^ (Canto et al., 2009). In current study, we observed that NOB effectively activated AMPK like hesperetin (data not shown). It raised a possibility that NOB may ameliorate inflammation in NASH by regulating AMPK or SIRT1 activity in immune cells in liver. To exclude this possibility, we treated LPS induced RAW 264.7 with Compound C (AMPK inhibitor) and EX-527 (SIRT1 inhibitor). In contrast to the re-elevation of inflammatory cytokines by RORα inhibitor SR3335, inhibition of AMPK and SIRT1 didn’t abolish the suppressive effect of NOB on the expression of these cytokines (Supplementary Figure 1), suggesting NOB induced AMPK activation was not involved in this process.

Among the polyphenols, in addition to NOB, there are other substances that have a regulatory effect on the polarization of macrophage. As a representative polyphenol, resveratrol-treated mice fed alcohol or a high-fat diet displayed preponderant M2 KC polarization, M1 KC apoptosis, and resistance to hepatocyte steatosis and apoptosis, as compared to control mice (Wan et al., 2014). Although the underlying mechanisms involved in NOB and resveratrol induced M2 polarization may be different, our current study combined with the previous study provide new perspectives to the exploration of anti-inflammatory mechanisms of polyphenols, especially in metabolic diseases associated chronic inflammation.

## Data Availability Statement

The original contributions presented in the study are included in the article/Supplementary Material, further inquiries can be directed to the corresponding author/s.

## Ethics Statement

The animal study was reviewed and approved by the Ethics Committee of Animal Experiments of Quzhou people’s hospital, China.

## Author Contributions

FZ and SW participated in research design. SW, TL, HS, FZ, ML, LW, HC, and CX conducted experiments. SW, TL, FZ, and HS performed data analysis. FZ and SW wrote or contributed to the writing of the manuscript. All authors contributed to the article and approved the submitted version.

## Conflict of Interest

The authors declare that the research was conducted in the absence of any commercial or financial relationships that could be construed as a potential conflict of interest.
