# Supplementary material for: Nobiletin Alleviates Non-alcoholic Steatohepatitis in MCD-Induced Mice by Regulating Macrophage Polarization
Source: Front Physiol. 2021 May 20;12:687744. doi: 10.3389/fphys.2021.687744 (PMC8174844; doi:10.3389/fphys.2021.687744)
Supplement: Supplementary file 1 [file Image_1.pdf]

Supplementary Material

Supplementary Figures

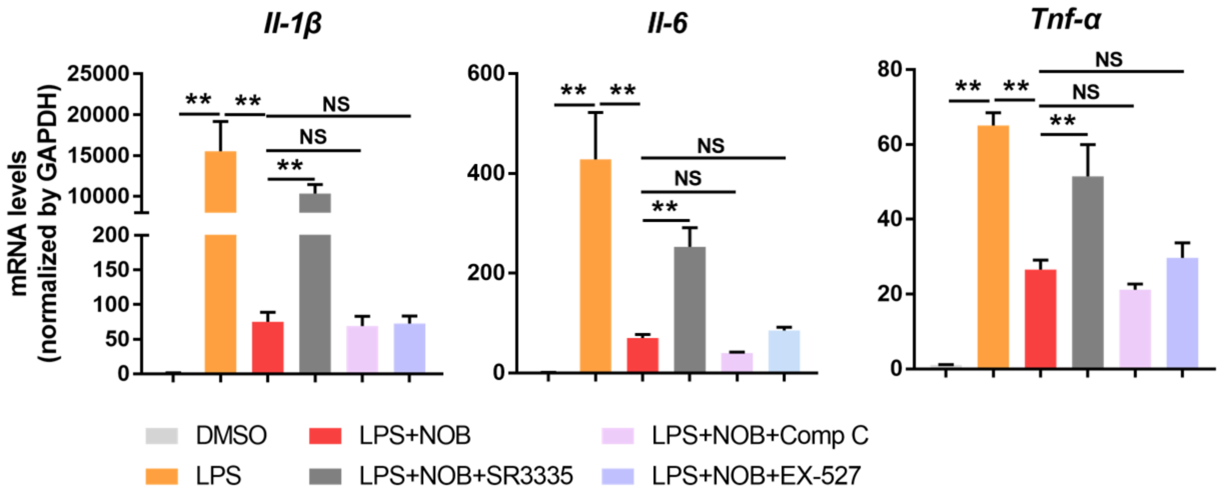

Supplementary Figure 1. The suppressive effect of NOB on the expression of pro-inflammatory cytokines was not affected by inhibition of AMPK and SIRT1. RAW 264.7 cells were pretreated with DMSO, LPS (200 ng/mL), LPS (200 ng/mL) + NOB (100 µM), LPS (200 ng/mL) + NOB (100 µM) + SR3335 (10 µM), LPS (200 ng/mL) + NOB (100 µM) + Compound C (Comp C, 100 nM, AMPK inhibitor), or LPS (200 ng/mL) + NOB (100 µM) + EX-527 (100 nM, SIRT1 inhibitor) for 16 h. Proinflammatory factors *Il-6*, *Il-1β* and *Tnf-α* determined by RT-qPCR. Values are expressed as mean± SD (n = 3). $^{**}P < 0.01$.
